# COVID-19 Severity and Cardiovascular Disease: An Inseparable Link

**DOI:** 10.3390/jcm11030479

**Published:** 2022-01-18

**Authors:** Michael Y. Henein, Matteo Cameli, Maria Concetta Pastore, Giulia Elena Mandoli

**Affiliations:** 1 Department of Public Health and Clinical Medicine, Umeå University, SE-901 87 Umea, Sweden; 2 Molecular and Clinic Research Institute, St. George London & Brunel Universities, London SW17 0QT, UK; 3 Department of Medical Biotechnologies, Division of Cardiology, University of Siena, 53100 Siena, Italy; matteo.cameli@yahoo.com (M.C.); pastore2411@gmail.com (M.C.P.); giulia_elena@hotmail.it (G.E.M.)

The COVID-19 pandemic is a global health issue that has so far affected over 250 million people worldwide. Having completed the first three waves of SARS-CoV2 infection, the world is currently facing the fourth wave, with significant consequences on overall global morbidity and mortality. The only effective proven weapons against COVID-19 are currently the vaccines and optimum prevention, in the form of personal protection, immune system strengthening with supplements, and personal isolation when tested positive. With this background, a thorough understanding of the viral mechanism of spread and underlying risk factors for critical disease is pivotal in order to limit COVID-19 detrimental consequences.

This Special Issue of the *Journal of Clinical Medicine* (JCM) entitled “Advances in Cardiology” offers four articles that contribute to the general physician and cardiologist’s knowledge on the role of cardiovascular risk factors and angiotensin-converting enzyme-2 (ACE-2) in COVID-19 severity.

It is known that the presence of cardiovascular diseases (CVDs) is associated with worse clinical conditions and higher mortality in patients who contract COVID-19. Data analysis of 44,672 patients with COVID-19 found that a history of CVD provided a nearly fivefold increase in fatality rates when compared with patients without CVD (10.5 vs. 2.3%) [1]. Among approximately 9000 patients hospitalized for COVID-19 in North America, Europe, and Asia, 30.5% had hyperlipidemia, 26.3% had arterial hypertension, 14.3% had diabetes mellitus, 16.8% were former smokers, and 5.5% were current smokers. Additionally, 11.3% had coronary artery disease, and 2.1% had congestive heart failure [2]. A meta-analysis of 48,317 patients with COVID-19 confirmed that CVD and cardiovascular risk factors are closely associated with fatal outcomes, irrespective of age [3]. Other meta-analyses have also shown that the prevalence of arterial hypertension or cardiac disease was >15% and was associated with a higher need for critical care management. Hypertension has been shown to induce a pre-activation of the immune cells, with raised inflammatory cytokines, which led to a surging immune response in patients in contact with SARS-CoV2 and delayed viral clearance. Moreover, patients with diabetes mellitus and COVID-19 infection were at a higher risk of admission to the ICU and mortality due to hyperglycemia-associated vascular endothelial cell dysfunction [4]. Obesity has also been shown to have a key role in COVID-19 infection severity, because of its known associated inflammation that contributes to the weakening of the immune system by augmenting adipose tissue production of pro-inflammatory cytokines and downregulating anti-inflammatory immune cells. 

Two of the studies published in this issue deal with a social category of people who are particularly at a high risk of exposure to SARS-CoV2 infection, i.e., clergy, because of the close inter-personal contact required during liturgies. Additionally, probably due to their special lifestyle, clergies are at significant risk because of the significantly high prevalence of CV risk factors they carry, including hypertension, diabetes, and obesity. The two published studies are part of our multicenter “COVID-CVD study”, which sought to investigate the importance of the various CV risk factors among Coptic clergies from Europe, the USA, and Egypt, in increasing their vulnerability to catching COVID-19, with its clinical consequences. In a group exceeding 1,600 clergies, the prevalence of SARS-CoV2 infection was 16.2%. Additionally, a model combining CV risk factors (hypertension, i.e., systolic blood pressure (SBP) ≥160 mmHg, diabetes mellitus, obesity, and history of coronary heart disease) was the most powerful independent predictor of COVID-19-related mortality, OR 3.991 ((1.919 to 6.844); *p* = 0.002) and the need for mechanical ventilation (OR 1.501 ((0.809 to 6.108); *p* = 0.001) [5]. In the second analysis, we found that obesity was the highest prevalent CV risk factor among Coptic clergies (above all, among Egyptians) and was the most powerful independent predictor of major COVID-19-related adverse events in the form of death or mechanical ventilation (OR = 4.180; 2.479 to 12.15; *p* = 0.01) [6]. These findings highlight the need for special attention to be given to clergy as a social category example for optimum protection from COVID-19 complications, with a serious need for lifestyle optimization and immune system support. Additionally, a well-designed education program for clergies is highly recommended, in order to optimally adhere to SARS-CoV2 contagion preventive measures and optimum control of CV risk factors according to available guidelines.

The Position Paper from VAS-European Independent Foundation in Angiology/Vascular Medicine for the Management of Patients with Vascular Disease or CV risk factors and COVID-19 suggested that lock-down policies for epidemic waves should target, in particular, patients with underlying CVD who should also undergo regular medical follow-up. They also recommended encouraging the use of telemedicine whenever possible, to improve adherence to antihypertensive, lipid-lowering, and hypoglycemic treatment according to current guidelines. It also recommends patients with underlying CVD and non-severe COVID-19 receive medical care at home, close follow-ups, and be prioritized for hospital admission when needed. 

With respect to anti-hypertensive treatment, the role of ACE-inhibitors has been extensively sought during the pandemic, since the aminopeptidase angiotensin-converting enzyme 2 (ACE2) has been identified as a receptor for SARS-CoV2, due to its binding to the spike protein of the virus. The review by Triposkiadis et al. [7], included in this issue, elucidates the role of ACE2 in COVID-19 progression and severity, reassuring the scientific community on the safety of continuing the use of ACE inhibitors.

In fact, ACE2 is highly expressed in many organs such as cardiomyocytes, enterocytes, renal tubular cells, and sinonasal cavity cells, whereas in the lungs, the ACE2 expression is minimum. However, the expression of ACE2 depends on the immune responses; therefore, binding of ACE2 with SARS-CoV2 may amplify inflammatory signaling and ACE2 expression, as well as promote virus replication, its entrance into the host cell, and its spread throughout the organism with the contribution of inflammatory M1 macrophages, which, in turn, have marked upregulation of ACE2 on their surface. Therefore, each action promoting the expression of ACE2 instead of its inhibition may be harmful to COVID-19 progression in the organism after SARS-CoV2 infection. The authors of this article also illustrate, in detail, the peculiar impact of COVID-19 and the role of the renin–angiotensin–aldosterone system in specific populations, such as patients with cancer, renal failure, and chronic obstructive pulmonary disease. Mechanism and disease-specific risk factors are described, such as tumor stage, disease progression, and type of cancer (above all thoracic), which are high-risk factors for disease severity in cancer patients. Again, the role of CV risk factors is highlighted with obesity and diabetes mellitus, resulting in an imbalance in the renin–angiotensin–aldosterone system, with higher ACE2 expression, which leads to slower viral clearance. In the end, emerging therapies targeting transmembrane protease, serine 2 (TMPRSS2), and ACE2 co-factor involved in the SARS-CoV2 binding and internalization are introduced, which represent new therapeutic frontiers against COVID-19.

The fourth article addresses an important in-hospital issue, which is the outcome of ST-elevation myocardial infarction, hence impacting the national health services and national health systems. Our hospital admission analysis showed a trend toward a reduction in acute coronary syndromes incidence, with a substantial increase in STEMI fatality rate and complications during the pandemic, compared with 2019 [8]. This was explained by the decrease in percutaneous coronary intervention (PCI) procedures [9], with an annual 634 PCI patients falling by 25.7% during the COVID-19 period (mean 30.0 ± 4.01 vs. 40.4 ± 5.3 case/month) and prolongation of the time from first medical contact to needle (125.0 ± 53.6 vs. 52.6 ± 22.8 min, *p* = 0.001). Such significant change in practice was interpreted on the basis of patients’ fear of visiting the hospital, lack of organized emergency pathways for acute coronary syndromes during the COVID-19 period, and occasional misdiagnosis in patients with respiratory symptoms. The last finding was higher in-hospital mortality (7.4 vs. 4.6%, *p* = 0.036), incidence of reinfarction (12.2 vs. 7.7%, *p* = 0.041), and the need for revascularization (15.9 vs. 10.7%, *p* = 0.046) during the COVID-19 pandemic. In fact, a dramatic increase in hospitalization for subacute myocardial infarction >72 h has been described worldwide, with increasing incidence of malignant arrhythmias and severe heart failure resistant to conventional therapy and often requiring inotropic or mechanical support. Untreated myocardial infarction is known to increase left ventricular maladaptive remodeling and the long-term incidence of dilated cardiomyopathy and heart failure. This would unavoidably constitute a clinical challenge and result in a poor prognosis. Possible solutions rely on optimal organization of healthcare services during the pandemic, social education, and alternative methods of follow-up to balance between the prevention of COVID-19 and acute coronary syndromes, such as the aforementioned telemedicine. 

Beyond coronary heart disease, since many cases were described as due to SARS-CoV2 infection or vaccine induced (the former with a 40-fold higher incidence than the latter), which may also be a potential confounder of an acute coronary syndrome—namely, acute myocarditis. In severe cases, these may cause life-threatening ventricular arrhythmias; therefore, arrhythmia monitoring may be crucial for these patients. Peretto G. et al. [10] conducted a study in 104 adult patients with biopsy-proven active myocarditis and de novo ventricular arrhythmias, who underwent prospective monitoring by both sequential 24 h Holter ECGs and continuous arrhythmia monitoring (CAM), including either implantable cardioverter defibrillator (ICD) (60%) or loop recorder (40%). The authors found that nearly half of the patients developed ventricular arrhythmias over long-term follow-up, CAM was more accurate in the identification of patients with ventricular arrhythmias, and histological signs of chronically active myocarditis (70%) and anteroseptal late gadolinium enhancement (25%) were significantly associated with the occurrence of ventricular tachycardia. These important findings may help the decision-making processes in patients presenting with acute myocarditis. 

Another consequence of myocarditis is the development of dilated cardiomyopathy (DCM), a complex disease for its variable etiology, complications, and management, irrespective of the etiology of DCM, primary, congenital/hereditary, or secondary, often a consequent to ischemic heart disease, myocarditis, infective or peripartum cardiomyopathy. Primary or secondary DCM may be complicated by valvular heart disease, chronic heart failure, arrhythmias, leading to sudden cardiac death; however, there are some primary forms that are particularly prone to develop arrhythmias, often presenting with sudden cardiac death, such as those deriving from LMNA gene (encoding for laminin A/C) mutation. The article by Ferradini et al. included in this issue [11] describes, among 77 families with DCM referred for genetic counseling and molecular screening, how they found 18 patients with heterozygotes mutation for laminin A/C with 2 new variants of the gene. Interestingly, 44.5% of patients presented with ventricular arrhythmias as the first symptom. These results highlight the importance of genetic analysis when laminin A/C mutation may be suspected in order to provide a good risk stratification of sudden cardiac death. In fact, there is currently a lack of targeted therapy for the treatment of LMNA variants-associated cardiomyopathy and the only therapy to consider is the prevention with ICD, also in these cases, because of the high risk of SCD in these patients.

It should be highlighted that ICD implantation is not free of risks. It carries a risk of pocket infection and leads to endocarditis with possible systemic infection, pneumothorax, or bleeding. It may also require new intervention either years after the first implantation, for example, to replace the battery, or earlier in the case of lead dislodging or for infections/malfunctions. While battery replacement without lead extraction is an almost simple procedure with only a few potential complications, transvenous lead extraction (TVE) is a challenging procedure that carries a high risk of life-threatening complications, such as superior vena cava tear, pericardial effusion, tamponade, and embolization. Therefore, a reconsideration of ICD indications is often operated when TVE should be performed, as recommended by the guidelines [12]. In this issue, D’Angelo et al. reported a study on 223 patients undergoing TVE, in 14.8% of whom no reimplantation was performed. At a median follow-up of 41 months, 11.8% received a new ICD after 17–84 months due to arrhythmic events. While hospitalization for device revision (in the reimplantation group) or late reimplantation (in the no-reimplantation group) was similar (11.1% vs. 12.1%, *p* = 0.771), as was short-term survival, five-year survival was significantly lower in the no-reimplantation group (78.3% vs. 94.7%, *p* = 0.014), and death occurred mostly for non-cardiac causes [13]. The absence of atrioventricular blocks in the primary indication and higher left-ventricular ejection fraction represented independent predictors favoring no-reimplantation. Therefore, these two elements may help therapeutic decisions, as these results recommend careful consideration of ICD reimplantation when TVE should be performed.

Finally, the eighth article in this Special Issue of Advances in Cardiology concerns a new but still important and timely topic, since the traditionally known non-alcoholic fatty liver disease, currently called metabolic-dysfunction fatty live disease (MAFLD), remains a challenging hepatic syndrome. Although its association with CV risk factors is well known, the mechanisms of its direct/indirect link with the CV system are still to be ascertained. In their article, Ismaiel et al. [14] investigated the association between adipokines, peptides product of the adipose tissue, and CV ultrasound parameters in 80 patients with hepatic steatosis evaluate by both hepatic ultrasonography and SteatoTestTM (40 patients with MAFLD diagnosis, 40 controls), who all underwent echocardiography and carotid Doppler ultrasound and adipokines analysis. The authors found that adiponectin and visfatin levels were not significantly different in MAFLD vs. controls. Visfatin was associated with mean carotid intima-media thickness, while adiponectin was associated with left ventricular ejection fraction and early/late diastolic waves (E/A) ratio in controls. A significant direct proportional association was found between adiponectin and E/A ratio in the univariate linear regression analysis but was lost in multivariate models. Conversely, although left ventricular ejection fraction was not significantly associated with adiponectin in univariate analysis, a significant inversely proportional association was demonstrated after adjustment using multivariate regression models, according to similar previous studies. These results may generate interesting hypotheses on the relationship between MAFLD and the CV system, but this needs to be further tested. 

In conclusion, the articles in this issue are expected to assist the readers confronted by COVID-19 patients and guide them to pay particular attention to CV risk factors and lifestyle. They also highlight the relationship between the COVID-19 pandemic and other cardiac syndromes, as well as its potential cardiovascular clinical consequences and their predictors, which, in some cases, could be avoided.
